# Clinical nurses’ compassion fatigue psychological experience process: a constructivist grounded theory study

**DOI:** 10.1186/s12912-023-01665-3

**Published:** 2023-12-19

**Authors:** Jie Zhang, Jie Zou, Xiao Wang, Yaoyue Luo, Jin Zhang, Zhiyao Xiong, Jingping Zhang

**Affiliations:** 1 Hunan University of Chinese Medicine, Changsha, China; 2 Hepatobiliary Pancreatic Cancer Center, Chongqing Key Laboratory of Translational Research for Cancer Metastasis and Individualized Treatment, Chongqing University Cancer Hospital, Chongqing, China; 3 The Third Xiangya Hospital of Central South University, Changsha, China; 4 General Ward of Musculoskeletal & Burn & Pain Rehabilitation Department, Department of Rehabilitation Medicine, West China Hospital, Sichuan University, Chengdu, China; 5 West China School of Nursing, Sichuan University, Chengdu, China; 6 Nursing Psychology Research Center of XiangYa School of Nursing, Central South University, 172 Tongzi Po Road, Changsha, Hunan 410013 China

**Keywords:** Clinical nurses, Compassion fatigue, Psychological experience, Constructivist grounded theory, Qualitative study

## Abstract

**Background:**

Clinical nurses are susceptible to compassion fatigue when exposed to various types of traumatic events in patients for extended periods of time. However, the developmental process, staging, and psychological responses distinct to each stage of compassion fatigue in nurses are not fully clarified. This study aimed to explore the processes of compassion fatigue and the psychological experiences specific to each phase of compassion fatigue among clinical nurses.

**Methods:**

Charmaz’s Constructivist Grounded Theory methodology was used in this qualitative research. Semi-structured interviews were conducted with 13 clinical nurses with varying degrees of compassion fatigue from December 2020 to January 2021. Interview data were analyzed using grounded theory processes.

**Results:**

The data were categorized into five separate categories and 22 sub-categories. This study found that the process of compassion fatigue is dynamic and cumulative, which was classified into five phases: compassion experience period, compassion decrement period, compassion discomfort period, compassion distress period, and compassion fatigue period.

**Conclusion:**

Clinical nurses who experience compassion fatigue may go through five stages that are stage-specific and predictable. The findings can shed light on local and global applications to better understand the problem of nurses’ compassion fatigue. The interventions for addressing compassion fatigue in clinical nurses should be stage-specific, targeted, and individualized.

**Supplementary Information:**

The online version contains supplementary material available at 10.1186/s12912-023-01665-3.

## Background

Compassion, as one of the core professional attributes of nursing care, is described as understanding another person’s suffering and trying to eliminate or alleviate it [1–3]. As professionals, nurses are expected to have empathy for their patients [4]. However clinical nurses have the most direct contact with patients and continuous exposure to patient pain, grief, and suffering, which put nurses at high risk of developing compassion fatigue [2]. Compassion fatigue has been recognized as an occupational hazard and has attracted scholars’ attention worldwide [5, 6]. Despite extensive literature that has explored nurses’ experiences of compassion fatigue, limited evidence demonstrates nurses’ compassion fatigue psychological experience process [7, 8]. Shedding light on this complexity would inform nursing practice and education globally.

Compassion fatigue describes the emotional, physical, and psychological weariness as a result of prolonged and intense work-related stress exposure [9]. Compassion fatigue in nursing refers to a cumulative and progressive process of absorbing patients’ pain and suffering that nurses experience as a result of their compassionate contact with patients and their families [10]. The conceptual structure of compassion fatigue proposed by Figley includes burnout and secondary traumatic stress [11], they both combine to raise the risk of compassion fatigue [12]. Compassion fatigue symptoms include headaches, insomnia, poor self-esteem, depression, tardiness at work, avoidance of work, and frequent sick leave [13]. Consequently, compassion fatigue can result in high turnover rates of nurses, decreased concentration and productivity, and even increased medication errors which ultimately affect patient satisfaction and safety [14, 15]. Compassion fatigue can happen to any nurse, at any time during the job course [10]. A meta-analysis including 21 studies found compassion fatigue is widespread among clinical nurses, with a prevalence of up to 52.55% [16]. Therefore, exploring the occurrence and development of compassion fatigue among nurses is warranted to effectively avoid compassion fatigue.

Nurses’ experiences can be thoroughly explored through qualitative research, which can then help us comprehend how they feel and behave when they suffer from compassion fatigue. After review of the literature revealed that current research on compassion fatigue in nurses mainly focuses on their experiences, facilitating factors, and coping strategies. For example, Pérez-García et al. [2] found that the lack of time and resources to provide comprehensive nursing care were facilitating factors of compassion fatigue. Compassion stress and overload can lead to compassion fatigue. Some strategies to reduce compassion fatigue are proposed, such as self-care, increased self-compassion, support and recognition from colleagues and family, and better work-life balance [7].

Based on the comprehensive literature review, we found that the current research about compassion fatigue among clinical nurses mainly focuses on the physical and psychological symptoms of compassion fatigue, facilitating and hindering factors, coping strategies and gains, etc. The processes of compassion fatigue and the psychological experiences specific to each phase of compassion fatigue among clinical nurses remain unknown, which brings obstacles to proposing targeted intervention programs for nurses in different stages of compassion fatigue. Therefore, this study aims to explore the processes of compassion fatigue and the psychological experiences specific to each phase of clinical nurses. We anticipate that the findings will be helpful and valuable in developing tailored, stage-matched intervention programs for clinical nurses.

## Methods

### Study design

A grounded theory methodology is a prospective approach to obtaining theoretical knowledge of psychosocial phenomena [17]. According to Charmaz’s constructivist grounded theory, the interaction between the researcher and the subject shapes the reality of the research findings. Because this interaction takes place within a broader social context, it highlights both the social construction process and the process of interaction between the two parties involved in the research [18]. This study attempts to articulate the psychological process of clinical nurses experiencing compassion fatigue and the psychological experiences specific to each stage, which is a reality formed in a specific historical and social developmental context, as well as an articulation and interpretation of the meanings co-constructed by the researcher and the participants. Charmaz’s constructivist grounded theory is highly compatible with the research purpose and content of the study, and therefore we chose this approach to guide this study. The Standards for Reporting Qualitative Research (SRQR) checklist was employed to present the results [19].

### Participants

Clinical nurses in a tertiary hospital in Changsha, China, were selected. Purposive sampling and theoretical sampling were used to choose the participants in accordance with the grounded theory approach’s guiding principles. At the beginning of the interview, purposive sampling was used. The inclusion criteria were the following: registered nurses in employment and working on the frontline, and nurses with different levels of compassion fatigue (mild, moderate, and severe) measured by the Professional Quality of Life Scale [20]. Stamm developed the original version [21], which was translated into Mandarin [22]. The scale was tested, and the findings demonstrated that it had strong validity and reliability and could be utilized to assess compassion fatigue among clinical nurses [23, 24]. The Chinese version of the professional quality of life scale has three dimensions: compassion satisfaction, burnout, and secondary traumatic stress [22]. The total score critical value of the above three dimensions is < 37 points, > 27 points, and > 17 points respectively [22]. If the total score of the three dimensions does not exceed the critical value, there is no compassion fatigue; if the total score exceeds the critical value of any dimension, it is mild compassion fatigue. If the total score of any two dimensions exceeds the critical value, it is moderate compassion fatigue, and if the total score of all three dimensions exceeds the critical value, it is severe compassion fatigue. Before the interview, 20 nurses were tested by the professional quality of life scale. Among them, 18 nurses had different levels of compassion fatigue and through objective sampling and theoretical sampling, finally, 13 nurses completed the interview. Data saturation was reached after 13 interviews when no new theoretical insights emerged as the information was analyzed.

### Data collection

Semi-structured interviews were conducted from December 2020 to January 2021. Prior to recruiting nurses, the researchers obtained consent from the directors and head nurses of each institution explaining the study’s purpose. Following the clinical nurses’ expression of interest in the study, an electronic version of the Professional quality of life scale was used to assess their level of compassion fatigue via Wenjuanxing (a Chinese online crowdsourcing platform). The principal researcher who was professionally trained in qualitative research contacted the participants by telephone and gave them a detailed introduction to the research content and purpose. Since this study was conducted at a time when COVID-19 was severe in China, phone interviews or video interviews through WeChat (a social networking software in China), relatively safe interview methods, were chosen to avoid the risk of infection to the participants and the researcher. The researcher established a trusting relationship initially by talking briefly with the interviewees before the formal interview, which proceeded smoothly because the primary researcher was a former clinical nurse and had many common topics. The researcher appropriately used techniques such as questioning, responding, verifying, summarizing, and silencing during the interviews to encourage the interviewees to express their inner feelings. The length of the interviews ranged from 30 to 70 min. Based on the literature review and pre-interviews, the researcher developed an interview guide, which included the following aspects: (1) feelings and experiences in the process of providing care to patients; (2) changes in feelings or experiences in the process of providing care to patients from the beginning to the present; (3) own awareness of compassion fatigue; (4) how to deal with compassion fatigue; (5) impressive cases in the process of experiencing compassion fatigue.

### Data analysis

Nvivo 11.0 was used to manage, store, and analyze the interview data. Data were systematically analyzed using initial coding, focused coding, theoretical coding, and constant comparative methods. Initial coding was carried out by continually “asking questions” of the original data through line-by-line coding and looking for nuances, explicit statements, and implicit meanings [17]. The initial codes with comparable meanings and features are then grouped together by ongoing comparison of the original codes, and the more pertinent and conceptual codes are then identified for focused coding [18]. The process of focused coding ultimately yielded five categories. By comparing, generalizing, and summarizing the codes and analogies, the continuous comparative analysis methodology is adopted throughout the study to comprehend the psychological experiences particular to each step and the process of clinical nurses’ compassion fatigue. The analysis was completed by the first author, who also consistently discussed it with the co-authors. Finally, the possibility of having preconceived notions about the study data was reduced by conducting a literature review pertinent to the research after the data analysis.

### Study rigor

The rigor of the study was accomplished in a variety of ways, including confirmability, credibility, and transferability [25]. To ensure confirmability, the researcher emailed the transcribed text to the participants and asked them to determine if the transcribed text was consistent with their views and experiences. In addition, the research team met frequently to discuss findings, plan future data collecting, examine transcripts, and evaluate data [26]. To ensure credibility, the research team accurately reviewed the interview transcripts, compared the extracted codes to the initial data, and repeatedly checked the analysis against the views of the study participants to maintain reflexivity and avoid the researcher’s views influencing the study data. To ensure transferability, a thick description strategy as a way of achieving a type of external validity was used [27]. The thick description strategy was used to describe not just the behavior and experiences, but their context as well [28]. For instance, nurses’ positive and enthusiastic work in this study was linked to their perception that their work will provide them with a feeling of achievement and that they are highly motivated to work every day.

## Results

**Fig. 1:**
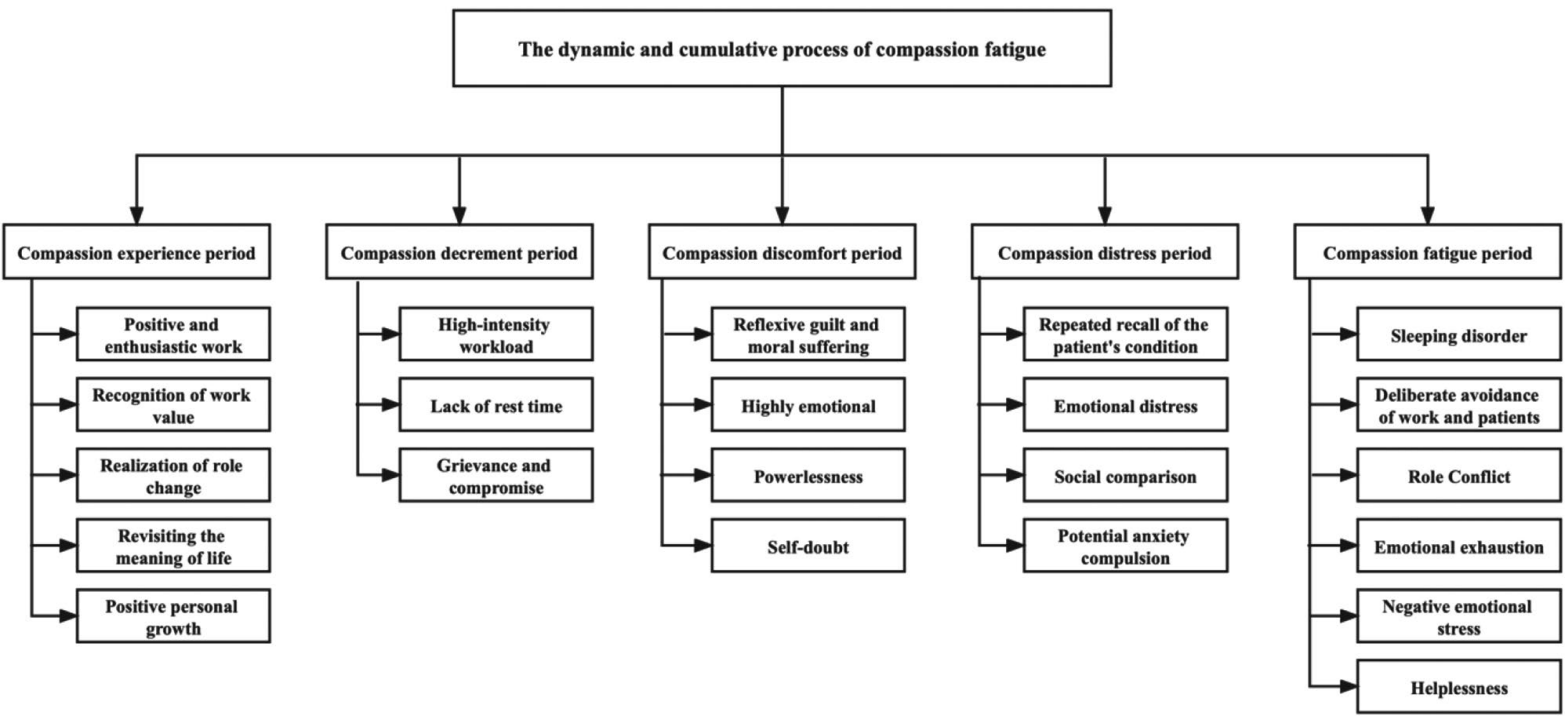
Stages and features of compassion fatigue development

**Fig. 2:**
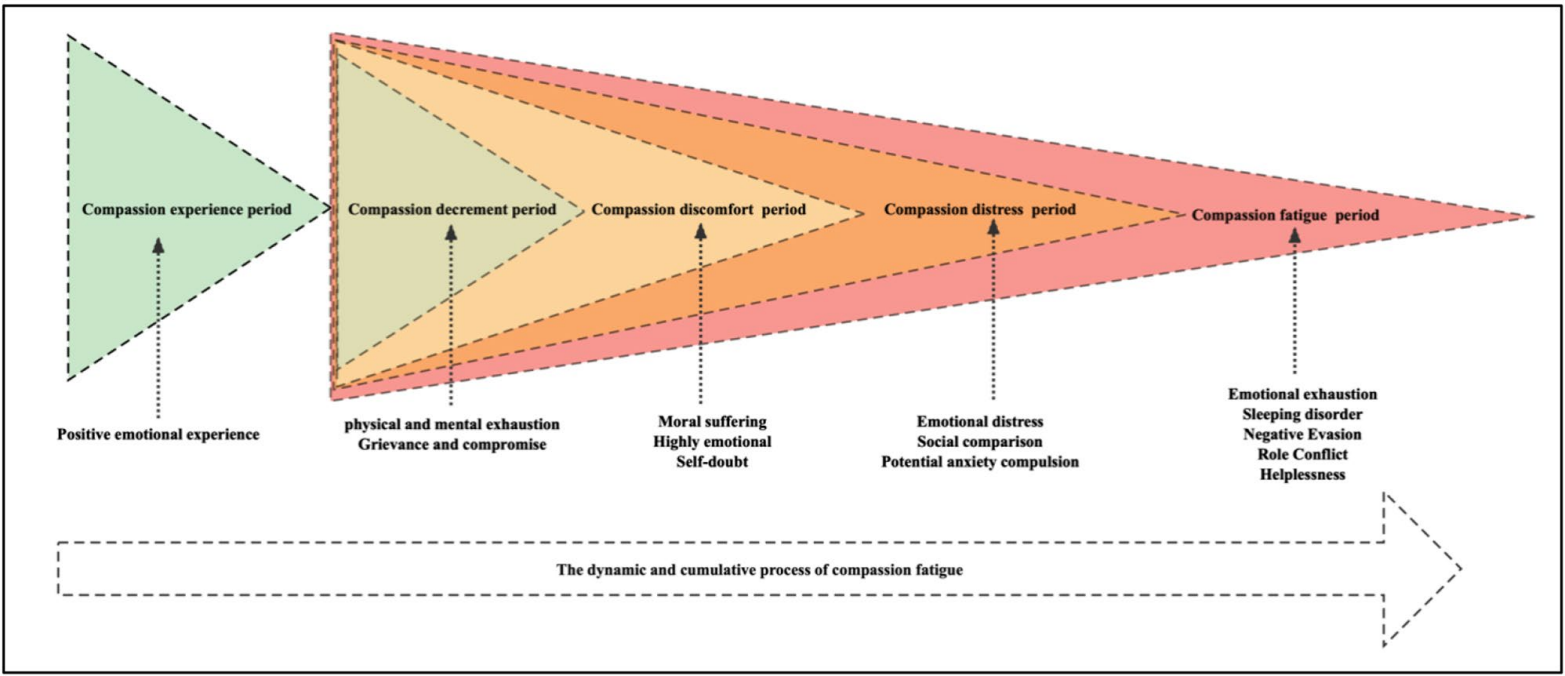
Schematic diagram of the developmental stages of compassion fatigue

The participants were all female, aged 23 to 46 years, with 3 to 24 years of experience; two of them had mild compassion fatigue, five had moderate compassion fatigue and six had severe compassion fatigue (Table 1). The data were categorized into five separate categories and 22 sub-categories (Fig. 1). This study found that the process of compassion fatigue is dynamic and cumulative, which was classified into five processes: compassion experience period, compassion decrement period, compassion discomfort period, compassion distress period, and compassion fatigue period (Fig. 2).

**Table 1:** Demographic characteristics of the participants

Number	Department	Sex	Age	Position	Education	Working Years	Level of compassion fatigue
N1	Spinal surgery	Female	31	Nurse	Master degree	9	Moderate
N2	Critical Care	Female	28	Nurse	Master degree	3	Severe
N3	Emergency	Female	28	Nurse	Bachelor degree	6	Severe
N4	Oncology	Female	36	Nurse	Bachelor degree	17	Moderate
N5	Head and neck surgery	Female	26	Nurse	Master degree	2	Severe
N6	Intervention	Female	35	Head nurse	Master degree	13	Severe
N7	Spinal surgery	Female	30	Nurse	College diploma	10	Moderate
N8	Hepatobiliary surgery	Female	23	Nurse	College diploma	4	Severe
N9	Hepatobiliary surgery	Female	28	Nurse	Bachelor degree	7	Moderate
N10	Respiratory Medicine	Female	29	Nurse	Bachelor degree	8	Severe
N11	Emergency	Female	30	Nurse	Master degree	4	Moderate
N12	Neurology	Female	36	Head nurse	Bachelor degree	14	Mild
N13	Emergency	Female	46	Head nurse	Bachelor degree	24	Mild

### Stage I: compassion experience period

#### Positive and enthusiastic work

When clinical nurses first entered clinical work, they were active and enthusiastic, full of expectations and hopes for the job, and more concerned about the condition of the patients under their charge.

*Because I love clinical work, I feel like every day after work, I look forward to going to work the next day, I am hopeful and motivated to do my job, and I want to do it well. (N5)*

Nurses will feel that their work will bring them a sense of accomplishment and that they are very motivated to work every day. When working they are passionate and treat their patients seriously and responsibly.

*When I first started, as a new nurse, I was full of energy, couldn’t stop, and thought my work was valuable. I enjoy the sense of accomplishment I get from helping patients. (N12)*

### Recognition of work value

In the process of helping patients, nurses experience a sense of satisfaction and accomplishment in their daily dealings with patients. In the process, they will take the initiative to give compassion, put themselves in the patient’s shoes to experience the patient’s state and needs, and meet their needs, thus gaining a sense of value and satisfaction.

*In my work, I can appreciate the patient’s inner needs and solve their problems in a timely manner, I think my work is quite meaningful and valuable. (N7)*

Nurses have an emotional contribution to caring for patients and receive affirmation from patients and families, which reflects the value of work and gives positive strength to nurses.

*I remember a patient in our department with an advanced tumor, but his family got along with us very well and his family was always grateful to us right after the patient died. Some of the family members also gave me positive energy (making me feel) that others remember me and recognize my work. (N3)*

### Realization of role change

Some nurses reported that they perceived their life and work to be relatively balanced and did not bring emotions at work into their lives. The transition between the role in life and the role at work was successfully achieved.

*I rarely bring emotions from the hospital into my life, and if I were to think back now, I wouldn’t be able to recall. (N9)*

Some nurses said that if they encountered difficulties or unhappiness at work, they would not bring what happened at work into their families, but adopt their own ways to digest and adjust, thus completing a successful transition of roles.

*I will use the ten minutes or so on the way to work, whether it is something at home or at work…I usually use ten minutes to complete the role change. (N12)*

### Revisiting the meaning of life

Clinical nurses gain a different perception and reflection of life and a greater reverence for life during their contact with patients.

*Our department is an oncology department, and many patients have to experience multiple chemotherapy treatments, which makes me think that people are stronger than they think. (N4)*

Clinical nurses reacquaint themselves with disease and the positive and negative aspects it brings through contact with patients in their regular work.

*My perception of the disease is not quite the same. I used to think that I would be more negative if I had cancer. But after the professional study and care of patients, I became more positive and optimistic. (N7)*

### Positive personal growth

Most of the nurses said that through the care of complex cases, they improved their working abilities and made progress in their professional skills.

*The most important thing is that I can learn and grow through work, whether it is learning to be or to do. (My work) exercises our logical thinking and enhances the rigor of the work, which can correctly establish my outlook on life and values. (N8)*

As clinical nurses gain experience, they become calmer and can help patients and families by utilizing what they have learned to ease their worries.

*After working for three or four years and gaining some work experience, I thought of taking care of patients beyond my skills, including providing psychological support and companionship to patients. (N12)*

### Stage II: compassion decrement period

#### High-intensity workload

Clinical nurses consider that their busy work schedule leaves them with no time to do what they love and that compassion-giving is slowly diminishing.

*I found myself wanting to do some things but simply did not have the time and energy to do them. I began to care less for my patients. (N2)*

Some clinical nurses said that their hectic clinical work will also accumulate some negative emotions and need time to adjust their negative emotions.

*Our work is fast-paced and without realizing it, we have accumulated some negative emotions there, and I need a break to adjust myself. (N7)*

### Lack of rest time

Clinical nurses have a lack of rest time and are physically exhausted, which is a major reason for their diminished compassion.

*Sometimes it was so busy that I went for a month without a break and even had more than 30 h without sleep before. Occasionally I still need to do (work-related) things after work, which makes me extremely fatigued. (N8)*

Matters outside of work sometimes take up a lot of rest time for clinical nurses, causing them to be physically and mentally exhausted.

*Every week we have difficult case studies and a lot of stuff that are just chores that take up a lot of my rest time and make me feel tired. (N5)*

### Grievance and compromise

Clinical nurses will give compassion, put themselves in the patient’s shoes and solve some problems for the patient or family, but if they do these things and the patient or family does not understand, the nurse feels frustrated and aggrieved. The enthusiasm for empathy will also be greatly reduced.

*Sometimes the family members may not understand, and they may speak viciously or violently to me, which (makes me) feel very grieved. (N3)*

Sometimes patients or families have a bias or disapproval of the nursing profession, which can also lead to diminished compassion for clinical nurses.

*I feel that sometimes I may be emotionally depressed because the patients as well as the families do not trust and affirm my dedication. (N12)*

### Stage III: compassion discomfort period

#### Reflexive guilt and moral suffering

In the workplace, clinical nurses are afraid to face the death of patients, expressing helplessness about their aggravation or passing away, and also harboring a sense of guilt and sadness.

*In the hematology department, there was a patient who was very close to me, but he still passed away, and it was a very sad time for me. (N8)*

Sometimes nurses feel guilty for not being able to help the patient, feeling frustrated that the patient is helpless, but that they are not able to help relieve their distress.

*(Sometimes I) look at patients and feel that they are helpless. I am also helpless because I feel a sense of frustration that I am not able to help patients in my workplace. (N13)*

### Highly emotional

Clinical nurses’ emotions are affected by the clinical outcome of patients, and they feel happy and accomplished if the patient has a good outcome. But if the patient has a poor clinical outcome, nurses will be sad and regretful.

*I feel sad when a patient is not resuscitated, and I think how could this happen, especially when some patients are very young…(N10)*.

It is easy for clinical nurses to put themselves in the position of the family and feel their emotions, leading to an inability to accept the passing of the patient.

*When we were at work (the patient) was still on the phone with his family and told them he was fine. As a result, the patient’s condition worsened and died in the afternoon. So how can the patient’s family accept (this result)? I can’t even accept it. (N3)*

### Powerlessness

Clinical nurses feel helpless and powerless when faced with patients, especially those whose conditions change rapidly and who suffer more pain.

*I can only say that I am so helpless, and then sometimes I want to cry when they (patients) cry. Because I can’t solve their problems. (N2)*

Clinical nurses can feel very helpless about the progression of a patient’s condition, and they can only do their best to make it better for them.

*I can’t control the patient’s condition, it’s just really helpless. I can only do my best to make them (the patients) feel better. (N2)*

#### Self-doubt

When clinical nurses make a significant effort for their patients, but their patients’ conditions do not improve significantly or even continue to deteriorate, they may doubt the value of their work at this time.

*Although I’ve been working for eight or nine years, I still can’t do that much when the patient is like this (when it’s serious). (N2)*

Clinical nurses sometimes have doubts about their abilities which may lead to depression.

*I was there (ICU) for three months, watching several patients die one after another, feeling that I could not do anything, at that time is a very powerless feeling, once doubted myself, I do this profession for what. There was a time of depression. (N7)*

### Stage IV: compassion distress period

#### Repeated recall of the patient’s condition

Clinical nurses will repeatedly recall the more impressive patients and their experiences after hours or in their lives, and subconsciously, they will impact their own lives.

*After a few days, I still think about the patient, including some things that I can still think about (the patient). (N10)*

Some clinical nurses said that they are also concerned about the change in patients’ condition after work and cannot control to know the condition of patients.

*Sometimes even when I am off duty, I worry about his (the patient’s) condition, and I take the initiative to ask my colleagues how the patient is doing. (N10)*

#### Emotional distress

During this phase, clinical nurses can experience difficulty in grasping the boundaries of emotions and can be infected by the emotions of the patient or family and brought into sadness.

*When I saw that scene (the patient died), I was sad, and then I saw the family crying, and I felt like crying too. (N5)*

Clinical nurses can suffer from more emotional distress due to excessive emotional commitment at work and can develop anxiety and bring that anxiety into their lives.

*(Our) work itself is intense because we have to keep our heads clear all the time, usually, this anxiety does not show up, but in life that anxiety, even depressed, negative emotions will reveal, I do not know why? (silent, emotional). (N11)*

#### Social comparison

Clinical nurses will analogize the patient’s condition and experience to their worry and fear.

*Because now there are more cars and car accidents, see them on which road accident, sometimes walking there from work, I will have a feeling of fear. (N13)*

Clinical nurses always have compassion for their patients and will internalize it in themselves and their families, and will caution their families to take care of their health.

*I still feel miserable about the patients, and of course, I sometimes think of myself. What should I do if my family becomes like this, I often remind my family members to take care of themselves. (N1)*

### Potential anxiety compulsion

Clinical nurses at this stage will have underlying anxiety and compulsive behavior. After work, they will reflect on whether the turnover of the patients they are in charge of was thorough and detailed, and they will frequently double-check.

*Sometimes after work, I worry about whether I’ve handed over my work clearly and whether I’ve missed anything. (N5)*

Sometimes the lack of professional competence causes clinical nurses to be cautious and overstressed in their work.

*I really feel that I am not capable and I am careful in everything I do, I am afraid that something will happen to that patient. (N2)*

### Stage V: compassion fatigue period

#### Sleeping disorder

Clinical nurses at this stage are often immersed in worry, and the invisible pressure of work causes the sleeping disorder.

*I worry every day …. and have nightmares almost every other day. (N3)*

The clinical nurse’s mind will go back and forth to the patient, the more insomnia, the more fear of going to work, and this vicious cycle occurs.

*I have an invisible pressure that causes me to have trouble sleeping, and I think about it repeatedly, and the more I can’t sleep, the more I repeat the cases of those patients in my mind. My insomnia is rather severe. (N2)*

### Deliberate avoidance of work and patients

Clinical nurses want to escape such a work environment and deliberately avoid contact with patients.

*I dread going to work every day. I want to postpone going to work and just accomplish what I must do now. I want to go (leave the hospital). (N8)*

Clinical nurses lack enthusiasm for their work and feel that their work is just numb work with no emotional input. They feel that they are not promoted in all aspects either, and will experience burnout and even want to leave their jobs.

*I feel like I’m not making any progress and I’m tired of working nonstop. Others claim that there is an incentive for work, yet I want to change my job. (N5)*

#### Role conflict

Clinical nurses present with a work-life imbalance that is impossible to achieve.

*Unpleasant things at home do not affect my work, but things at work affect my life, probably because I regard my work as more important. (N6)*

*After working for so long, I still feel that I will bring some emotions from work into my life, and I feel that I can’t distinguish between life and work. (N3)*

#### Emotional exhaustion

Having experienced numerous patients in a wide variety of situations, clinical nurses can become numb and calm.

*Overall, I’m starting to get numb to some things at work. (N10)*

Clinical nurses have seen many sad scenes, after which they feel used to them and take them for granted without much feeling.

*I have experienced a lot of resuscitation, experienced too many times, and got used to it, then I became not have too much feeling, a little numb. (N10)*

## Negative emotional stress

Clinical nurses develop a sense of depression and fatigue.

*My character is more serious, so when I unknowingly give more of myself emotionally, it is simple to walk in that mood. Sometimes, I even worry that I’ll carry that emotion home, which causes me to feel anxious and depressed (Silent crying). (N7)*

### Helplessness

Clinical nurses can become helpless, with nowhere to go to relieve their fears and anxieties, feeling that some people will not understand their state and not knowing whom to turn to for help.

*I don’t know where to express my bad emotions, and I feel helpless. I’m not even able to mention these things to some people since they could not understand you and even believe you can’t handle the suffering. (N2)*

## Discussion

Our study outlined the stages of compassion fatigue and its characteristics in clinical nurses. The whole process of compassion from the initial giving of compassion to the gradual development of compassion fatigue was demonstrated. Coetzee et al. [29] after a comprehensive literature review outlined the process of compassion fatigue as a progressive and cumulative process, from compassion discomfort to compassion stress to compassion fatigue, which is partially consistent with the results of this study. Our research expanded and enriched with a more detailed division into five stages, including the compassion experience period, compassion decrement period, compassion discomfort period, compassion distress period, and compassion fatigue period.

In this study, nurses at the beginning of their careers experience a period of compassion characterized by positive and enthusiastic work, recognition of work value, realization of role change, revisiting the meaning of life, and positive personal growth. Mei et al. [30] indicated that in the initial stage of nurses demonstrating compassion, they experience positive emotions like a sense of self-worth and professional accomplishment, which is in line with the results of this study. In addition, Berg et al. [31] interviewed trauma group members and the results of the study also showed that they would be motivated to work initially, further supporting the findings of this study. For this stage, we can fully explore its positive factors to actively maintain the experience during this period. Klein et al. [5] conducted a study implementing a resilience program focused on education about compassion fatigue, hoping to develop an ongoing self-care practice to prevent compassion fatigue. In other words, clinical nurses can preserve the period of compassion by utilizing their individual psychological resources (e.g., psychological resilience, self-compassion, and optimism).

With the increase in workload and lack of rest time, nurses will feel physically exhausted. Coupled with the fact that their emotional commitment is not understood by patients and families, nurses will feel aggrieved, thus entering a period of compassion decrement. This is in accordance with the findings of a previous study on the emotional experience of compassion fatigue in emergency department nurses [32]. In this period, the patients’ and family members’ lack of understanding will diminish the nurse’s compassion experience due to the increased workload and lack of rest time. At this time, the key point of intervention is to consider seeking social support from society, hospitals, and families. At the hospital level, we can consider a rational allocation of human resources and a moderate reduction of work intensity; at the family level, family and friends can give more support and assistance; at the social level, we are actively guiding through the media to create a positive professional atmosphere for nurses.

Following failed treatment of patients, nurses go through a time of compassion discomfort brought on by psychological guilt and self-blame, emotions of helplessness, intense emotional misery, and unseen moral suffering. Studies have found that when nurses become overly empathically devoted to their patients, the slight discomfort or distress of patients can elicit a strong response from the nurse due to excessive emotional and responsible involvement [7, 33]. During times of compassion discomfort, clinical nurses generally feel moral distress and self-doubt. The self-efficacy theory of Bandura contends that behavior and self-efficacy are strongly related [34]. At this point, nurses have self-doubt about their competence, so increasing self-efficacy might improve their psychological well-being and consequently, their behavior. Verbal encouragement, alternative experiences, and behavioral performance successes serve as the foundation for an individual’s sense of self-efficacy [34]. Therefore, researchers can raise nurses’ sense of self-efficacy through the power of role models, encouraging words, and feedback to pass through the compassion discomfort period among clinical nurses.

The emotional distress of clinical nurses is exhibited in the compassion distress phase. Nurses will repeatedly focus on and recall things related to the patient, and this repeated recall has disrupted the nurses’ lives, disturbed them emotionally, and can create an underlying sense of anxiety and compulsion in their lives. In addition, this study found that nurses will socialize the comparison from the patient’s events and will analogize the patient’s condition and experience to themselves, to worry and fear, and occasionally experience fear of illness. During this time, nurses will be influenced by patients’ emotional responses and may experience psychological pain. They may likely have anxiety and obsessive symptoms in life if they keep thinking about the patients’ circumstances. The pivotal point of intervention at this stage can be considered to use cognitive restructuring, which transforms negative, self-defeating thinking into positive, self-affirming notion.

As negative emotions accumulate, nurses eventually encounter a phase of compassion fatigue. Numbness and emotional exhaustion in the emotional experience, avoidance and alienation from work behavior, and physical problems including sleep disorders all occur, which is corresponding with results from previous studies [2, 7]. If the nurse experiences physical problems, such as sleeping difficulties, they may seek medical assistance and prescription medications. The psychological effects of emotional exhaustion can be alleviated through active interventions including emotional catharsis, attention shifting, and focusing on a spiritual outlet (such as religious belief) to help recovery [7].

### Relevance for clinical practice

Compassion fatigue in clinical nurses develops over time and is a dynamic process, which can shed light on a better understanding of the problem of nurses’ compassion fatigue locally and globally. The findings contribute to nursing management and education in the field of solving compassion fatigue in clinical nurses. The implications for nursing management include that clinical nurses’ experience of compassion fatigue is a cumulative process. Interventions for compassion fatigue in clinical nurses should be staged, targeted, and individualized to maintain the compassionate experience period, pass through the compassionate discomfort period, cope with the compassion fatigue period, and ultimately reduce psychosomatic damage among clinical nurses.

In terms of nursing education, these findings urge consider teaching nursing students and new nurses about the development of compassion fatigue so they may be prepared for the problems they may encounter in the future. The majority of the existing intervention programs are designed to help nurses who are already suffering from compassion fatigue. Instead, we should emphasize prevention more, and a cost-effective way to do this is by creating educational programs to raise clinical nurses’ awareness of and knowledge about compassion fatigue.

#### Limitations

The shortcomings of this study include the following aspects. Firstly, due to the impact of COVID-19, the study subjects were interviewed by telephone or video, and there may be an inability to carefully observe the participants’ body language and facial expressions during the interview process. However, throughout the study, the researcher deeply felt that the participants were more willing to confide their feelings through the long-distance interviews with electronic devices, providing a wealth of information that could help the researcher understand more deeply the emotional experience during the process of compassion fatigue. Secondly, all the interviewees were female, and there may be differences in the feelings of male nurses; however, most of the nurse population was female, and the results of this study may also inform the psychological experience of the developmental process of compassion fatigue for most nurses; thus, further studies conducted with male nurses are needed.

## Conclusions

Clinical nurses who experience compassion fatigue may go through five stages that are stage-specific and predictable: compassion experience period, compassion decrement period, compassion discomfort period, compassion distress period, and compassion fatigue period. This study pointed out a noteworthy issue regarding the significance of tackling compassion fatigue based on the characteristics of different stages. The analysis showed that if prompt intervention is not given to clinical nurses during the early stages of compassion fatigue, such as the periods of compassion decrement, compassion discomfort, and compassion distress, they will eventually enter a period of compassion fatigue, which is especially harmful to the nursing profession building on compassion and caring. This study critically advocates for hospital administrators to better address this issue by creating customized, stage-appropriate intervention programs.

### Electronic supplementary material

Below is the link to the electronic supplementary material.

Supplementary Materials: Standards for reporting qualitative research (SRQR)

## Data Availability

Due to ethical concerns from the perspective of the participants, the datasets generated and/or analyzed during the current study are not publically available, but they are available from the corresponding author upon justifiable request.

## Abbreviations

SRQR: Standards for reporting qualitative research
COVID-19: Corona virus disease 2019
